# Nonsense variants of *STAG2* result in distinct congenital anomalies

**DOI:** 10.1038/s41439-020-00114-w

**Published:** 2020-09-18

**Authors:** Hiromi Aoi, Ming Lei, Takeshi Mizuguchi, Nobuko Nishioka, Tomohide Goto, Sahoko Miyama, Toshifumi Suzuki, Kazuhiro Iwama, Yuri Uchiyama, Satomi Mitsuhashi, Atsuo Itakura, Satoru Takeda, Naomichi Matsumoto

**Affiliations:** 1 Department of Human Genetics, Yokohama City University Graduate School of Medicine, Yokohama, Japan; 2 Department of Obstetrics and Gynecology, Faculty of Medicine, Juntendo University, Tokyo, Japan; 3 Department of Obstetrics and Gynecology, Koshigaya Municipal Hospital, Saitama, Japan; 4 Department of Neurology, Kanagawa Children’s Medical Center, Kanagawa, Japan; 5 Department of Neurology, Tokyo Metropolitan Children’s Medical Center, Fuchu, Tokyo, Japan

**Keywords:** Disease genetics, Next-generation sequencing

## Abstract

Herein, we report two female cases with novel nonsense mutations of *STAG2* at Xq25, encoding stromal antigen 2, a component of the cohesion complex. Exome analysis identified c.3097 C>T, p.(Arg1033*) in Case 1 (a fetus with multiple congenital anomalies) and c.2229 G>A, p.(Trp743*) in Case 2 (a 7-year-old girl with white matter hypoplasia and cleft palate). X inactivation was highly skewed in both cases.

## Introduction

Cohesin is a multisubunit protein complex consisting of four core proteins: structural maintenance of chromosome 1 (SMC1), structural maintenance of chromosome 3 (SMC3), RAD21 cohesin complex component (RAD21), and stromal antigen (STAG)^1^. The cohesion subunit STAG1, STAG2, or STAG3 can directly attach to a tripartite ring (comprising SMC1, SMC3, and RAD21) to entrap chromatids^1^. Other interacting proteins, such as the cohesin loader NIPBL, also regulate the biological functions of cohesion^1^.

Cohesin is involved in a range of important functions, including functions in sister chromatid cohesion, DNA repair, transcriptional regulation, and architecture^1,2^. Hence, germline pathogenic variants of genes encoding cohesin subunits and their interacting proteins, such as *NIPBL*, *SMC1A*, *SMC3*, and *RAD21*, are known to cause developmental disorders referred to as cohesinopathies^3^, and these are characterized by intellectual disability (ID), growth retardation, and limb abnormalities^4^.

Recently, *STAG2* was added to the list of genes mutated in cohesinopathies^5,6^. As STAG2 is essential for DNA replication fork progression, STAG2 defects may result in replication fork stalling and collapse with disruption of the interaction between the cohesin ring and the replication machinery as previously described^7^. To date, 16 pathogenic variants of *STAG2* have been reported, including seven nonsense, four missense, one splicing, and four frameshift variants^5,8–13^. Notably, seven male patients in three families harbored missense variants. In one family, five affected males showed ID and congenital abnormalities^11^, and two other sporadic males were reported to have dysmorphic features, short stature, hypotonia, developmental delay (DD) and ID^9,10^. Female patients had truncated and missense variants^5,8,10,12,13^. Here, we describe the genetic and clinical features of two female cases with de novo nonsense variants of *STAG2*.

Case 1 was the second conceptus of healthy Japanese nonconsanguineous parents (a 35-year-old mother and 37-year-old father). At 15 gestational weeks, holoprosencephaly, cleft palate, cleft lip, blepharophimosis, nasal bone absence, and hypolastic left heart were noted by ultrasonography. The fetal karyotype determined by amniocentesis at 18 gestational weeks was normal (46,XX). The pregnancy was terminated at 21 gestational weeks because of multiple fetal abnormalities.

Case 2 was a 7-year-old girl who was born as the second child to healthy nonconsanguineous parents. She was born uneventfully at full term. Her birth weight was 2734 g (–1.3 SD). A cleft palate was noted at birth and surgically repaired at 1 year. She presented with mild dysmorphic features, including a long philtrum. At 8 months, she developed afebrile convulsions for which carbamazepine was effective. Anticonvulsants were discontinued at 4 years with no subsequent attacks. She acquired independent gait at 2 years and spoke only a few words at 7 years. Brain magnetic resonance imaging at 7 years revealed white matter hypoplasia. She currently has mild DD, ID, sensorineural hearing loss, and amblyopia with no neurologic abnormalities. She attends a school for hearing-impaired children.

This study was approved by the institutional review board of Yokohama City University School of Medicine. WES was performed in the two cases (Cases 1 and 2) and their parents. Blood leukocytes from the patient (Case 2), parents (Cases 1 and 2) and umbilical cord (Case 1) were obtained after obtaining informed consent. Exome data acquisition, processing, annotation, and filtering and variant calling were performed as previously described^14^. Possible pathogenic variants were evaluated based on mutational type (nonsense, missense, frameshift, or splice site) using the SIFT score (http://sift.jcvi.org/), Polyphen-2 (http://genetics.bwh.harvard.edu/pph2/), Mutation Taster (http://MutationTaster.org/), and CADD (https://cadd.gs.washington.edu/). Possible pathogenic variants were validated by Sanger sequencing. Parentage was confirmed using 12 microsatellite markers with Gene Mapper software v4.1.1 (Life Technologies Inc., Carlsbad, CA).

Total RNA was extracted from lymphoblastoid cell lines (LCLs) with the RNeasy Plus Mini Kit (Qiagen, Germany) and, reverse-transcribed to cDNA with the Super Script First Strand Synthesis System (Takara, Japan), and the cDNA used as templates for RT-PCR. PCR amplicons were subjected to Sanger sequencing.

CNVs were examined using WES data by two algorithms: the eXome Hidden Markov Model^15^, and a program based on the relative depth of coverage ratios developed by Nord et al.^16^.

X chromosome inactivation was determined using the human androgen receptor gene. X-inactivation ratios (expressed arbitrarily as a ratio of the smaller allele to the larger allele) were calculated twice and judged as published criteria: <20:80 (random), >20:80 (skewed), and >10:90 (highly skewed)^17^.

Ten micrograms of sheared DNA was subjected to library preparation using a single-molecule real-time (SMRT)bell Express Template Prep Kit 2.0 (Pacific Biosciences, 100-938-900) and a SMRTbell Enzyme Cleanup Kit (Pacific Biosciences, 101-746-400) in accordance with the manufacturer’s instructions (Procedure & Checklist - Preparing HiFi SMRTbell® Libraries using SMRTbell Express Template Prep Kit 2.0, Pacific Biosciences). One SMRT cell was used for the patient (Case 1). Secondary analysis using base-called data was performed using SMRT analysis v8.0 (Pacific Biosciences). Circular consensus sequencing (CCS) from single molecules was performed, and the generated sequence was mapped to the hg19 human reference genome using the CCS with Mapping application, provided by SMRT analysis, with the default settings. DeepVariant 0.9.0 (https://github.com/google/deepvariant) was used to detect SNVs and indels in CCS reads. The aligned CCS BAM data from the CCS with Mapping application were used as an input. We ran Google DeepVariant with a model trained for PacBio CCS (--model_type=PACBIO) using the prebuilt Docker image from the DeepVariant public repository (https://github.com/google/deepvariant). Small variant calls from DeepVariant were haplotyped and phased using WhatsHap 0.18 (https://whatshap.readthedocs.io/en/latest/).

**Fig. 1:**
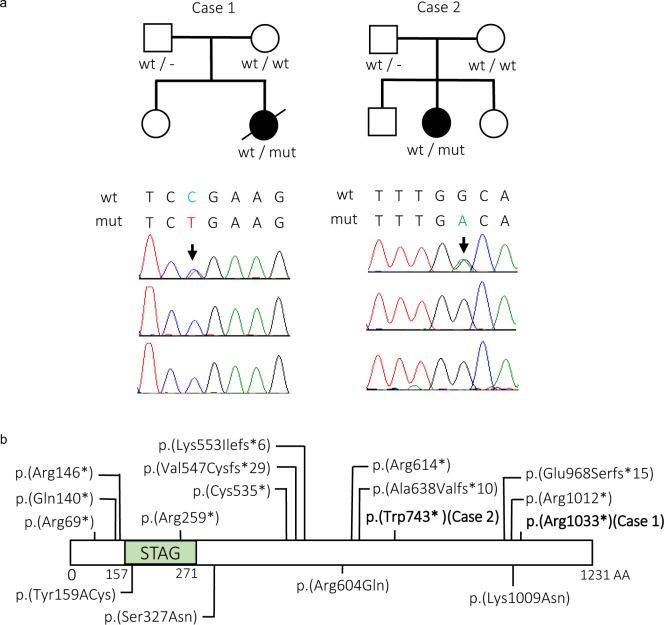
Summary of pathogenic variants of *STAG2*. **a** Familial pedigrees and electropherograms of *STAG2* variants [Case 1: c.3097 C>T: p.(Arg1033*), Case 2: c.2229 G>A, (p.Trp743*)]. The arrow indicates a heterozygous variant. wt, wild-type; mut, mutation. **b** Functional domain of the STAG2 protein and pathogenic variants. Truncating and missense variants are shown above and below the protein, respectively. Our cases are shown in bold. The STAG domain predicted by Pfam is shown (http://pfam.xfam.org).

**Fig. 2:**
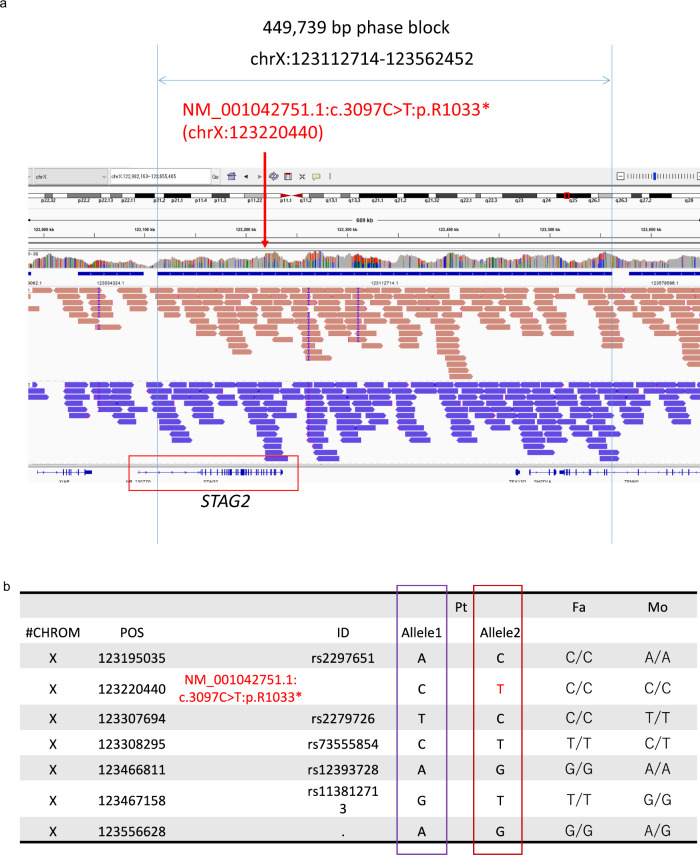
Confirmation of the pathogenic *STAG2* variant in the paternal chromosome in Case 1. **a** The c.3097 C>T variant could be successfully mapped within the 450-kb phased haplotype block in Case 1 using HiFi sequence and haplotype phasing. **b** SNP typing confirmed that the mutation occurred in the paternal chromosome (Allele 2, the brown haplotype block in Fig. 2a). POS: position of sequence, Pt: patient, Fa: father, Mo: mother.

We first performed WES in Case 1. Case 1 had no pathogenic variants in 14 known mutated genes associated with holoprosencephaly, namely, *SHH*, *ZIC2*, *SIX3*, *TGIF1*, *GLI2*, *PTCH1*, *DISP1*, *FGF8*, *FOXH1*, *NODAL*, *TDGF1, GAS1*, *DLL1*, and *CDON*. Moreover, no pathogenic CNVs were identified by exome-based CNV analysis. After analyzing trio-based WES data, three de novo variants were found. (Supplementary Table S1), but two missense variants were likely benign based on computational predictions. The remaining de novo nonsense variant [c.3097 C>T, p.(Arg1033*)] of *STAG2* was confirmed by Sanger sequencing (Fig. 1a) and was likely causative. X inactivation was highly skewed (93:7), and the paternal X chromosome was inactivated (Supplementary Fig. S1). Unfortunately, living cells from Case 1 could not be obtained for further mRNA analysis. Using HiFi long-read genome sequencing and haplotype phasing with informative variants, we constructed haplotypes in the vicinity of *STAG2* and confirmed that the *STAG2* variant occurred de novo on the paternal chromosome in Case 1 (Fig. 2). As the paternal X chromosome is mostly inactivated in blood leukocytes, the X inactivation pattern should be favorable in Case 1. We also identified another *STAG2* nonsense mutation [c.2229 G>A, p.(Trp743*)] occurring de novo in Case 2 (Fig. 1a, Table 1). X inactivation was highly skewed (96:4), and the maternal X chromosome was inactivated (Supplementary Fig. S1). RT-PCR indicated that only the wild-type allele was expressed in LCLs of Case 2 (Supplementary Fig. S2). Even after cycloheximide treatment, the mutant allele was completely undetectable, suggesting that it was transcriptionally repressed (through favorably skewed X inactivation) rather than posttranscriptionally diminished (through nonsense-mediated mRNA decay) in cultured LCLs. Regardless of the favorable X inactivation pattern in both cases, Case 1 was clinically much more severe than Case 2. Therefore, it is difficult to discuss phenotype severity in relation to the X inactivation pattern.

**Table 1:** Clinical features of patients with *STAG2* variants.

Characteristics	Case 1 This study	Case 2 This study	Mullegama et al.^7^		Lan Yu et al.^5^			Yuan et al.^9^		
Sex	Female	Female	Female	Female	Female	Female	Female	Female	Female	Male
***STAG2*** **variants**	c.3097 C>T	c.2229 G>A	c.205 C>T	c.1913_1922 del	c.1840C>T	c.418 C>T	c.1605T>A	c.1658_1660 delinsT	c.1811G>A	c.476 A>G
**Protein change**	p.(Arg1033*)	p.(Trp743*)	p.(Arg69*)	p.(Ala638Valfs*10)	p.(Arg614*)	p.(Gln140*)	p.(Cys535*)	p.(Lys553Ilefs*6)	p.(Arg604Gln)	p.(Tyr159Cys)
**Inheritance**	de novo	de novo	de novo	de novo	de novo	de novo	de novo	de novo	de novo	de novo
**Microcephaly**	NA	−	+	NA	+	−	+	+	+	−
**Brain MRI/US findings**	HPE	white matter hypoplasia	dysgenesis of the splenium of the corpus callosum, subarachnoid cyst, subgaleal hematoma	NA	cystic pituitary lesion	NA	NA	HPE (microform)	NA	ectopic posterior pituitary, short pituitary stalk
**Dysmorphic features**	NA	+	+	NA	NA	+	+	+	+	+
**Craniofacial anomalies**	cleft lip/palate	cleft palate	cleft palate	NA	NA	−	−	−	NA	cleft lip/ palate
**Congenital heart defects**	left heart hypoplasia	−	VSD	NA	NA	left heart hypoplasia, VSD, CA	NA	NA	−	minimal PFO
**Thoracic vertebra anomalies**	NA	hemi vertebra	scoliosis, hemivertebra, butterfly vertebra	NA	scoliosis	scoliosis, rib fusion, vertebral clefts	−	scoliosis, rib fusion	vertebral clefts	scoliosis
**Developmental delay**	NA	+	+	NA	+	+	+	+	+	+
**Intellectual disability**	NA	+	+	NA	NA	NA	+	+	+	+
**Other features**		amblyopia, seizures	left facial palsy, mild left pelviectasis		sacral dimple, CDH	gastro-esophageal reflux, CDH seizures	hypotonia	seizures, hypotonia	gastro-esophageal reflux, CDH, pulmonary hypoplasia, hypotonia	single kidney, hypotonia

Characteristics	Kruszka et al.^11^						Berkovic et al.^12^	Mullegama et al.^8^	Soardi et al.^10^
Sex	Female	Female	Female	Female	Female	Female	Female	Male	Male
***STAG2*** **variants**	c.205 C>T	c.436 C>T	c.775 C>T	c.3034 C>T	c.2898_2899del	c.2533 + 1 G>A	c.1639delG	c.3027 A>T	c.980 G>A
**Protein change**	p.(Arg69*)	p.(Arg146*)	p.(Arg259*)	p.(Arg1012*)	p.(Glu968Serfs*15)		p.(Val547Cysfs*29)	p.(Lys1009Asn)	p.(Ser327Asn)
**Inheritance**	de novo	singleton	de novo	de novo	de novo	maternally	de novo	de novo	maternally
**Microcephaly**	+	+	−	+	+	+	NA	+	0/5
**Brain MRI/US findings**	HPE (semi-lobar)	HPE (alobar)	HPE (septo-optic dysplasia)	HPE (alobar)	HPE (microform)	HPE (semi-lobar)	+	normal at 1 year	NA
**Dysmorphic features**	+	+	−	+	NA	−	+	+	5/5
**Craniofacial anomalies**	cleft palate, micrognathia	cyclopia, absent nose, hypognathia	−	cleft lip/palate	NA	−	NA	−	cleft palate (1/5)
**Congenital heart defects**	PFO, PDA	VSD	VSD	NA	−	left heart hypoplasia, DORV	NA	−	0/5
**Thoracic vertebra anomalies**	−	hemi vertebra	NA	spina bifida	NA	NA	NA	−	NA
**Developmental delay**	+	NA	+	NA	+	NA	+	+	0/5
**Intellectual disability**	NA	NA	+	NA	NA	NA	NA	+	5/5
**Other features**		duodenal atresia	left hip dysplasia, bilateral optic nerve hypoplasia	gastro-esophageal reflux			seizures, hearing loss	hypotonia	

c.980 G > A was reported in a family with five affected males.

*NA* not available, *MRI* magnetic resonance imaging, *US* ultrasonography, *HPE* holoprosencephaly, *VSD* ventricular septal defect, *CDH* congenital diaphragmatic hernia, *CA* coarctation of the aorta, *PFO* patent foramen ovale, *PDA* patent ductus arteriosus, *DORV* double-outlet right ventricle.

In addition to the two variants in our cases, a total of 16 pathogenic variants of *STAG2* have been reported in unrelated families (Table 1), including 12 truncated variants [p.(Arg69*), p.(Gln140*), p.(Arg146*), p.(Arg259*), p.(Cys535*), p.(Val547Cysfs*29), p.(Lys553Ilefs*6), p.(Arg614*), p.(Ala638Valfs*10), p.(Glu968Serfs*), p.(Arg1012*), and c.2533+ 1 G] and four missense variants [p.(Tyr159Cys), p.(Ser327Asn), p.(Arg604Gln), and p.(Lys1009Asn)]^5,8–13^.

For a female patient with p.(Ala638Valfs*10), no detailed phenotype was provided in the DECIPHER database, therefore, this patient was omitted for further comparison of clinical features^8^. Twelve cases with *STAG2* truncation variants reported in the literature were all females, and one missense variant was reported in a female patient. The 12 female cases with *STAG2* truncation shared microcephaly (10/12), abnormal brain MRI findings (10/12, including holoprosencephaly 7/12), thoracic vertebral anomalies (6/12), DD (9/12), and ID (4/12). Case 1 showed severe clinical features, such as holoprosencephaly and hypoplastic left heart, similar to previous literature, while Case 2’s clinical features were relatively mild. p.(Arg69*) was recurrent in two unrelated patients, both showing middle brain anomalies^8,12^.

Our two cases showed highly skewed X inactivation (93:7 in Case 1 and 96:4 in Case 2) (Supplementary Fig. S1). To date, X inactivation analysis has been reported in only two cases, one with skewed X inactivation and another with random X inactivation, but the exact ratios were not shown in the literature^10,12^. Interestingly, one splicing variant (c.2533+ 1 G) was inherited from the mother, but unfortunately, an X inactivation study was not conducted^12^.

In contrast, null *STAG2* variants in males have never been reported. We speculate that males with a hemizygous truncating *STAG2* aberration are lethal or show severe fetal clinical ends. Interestingly, one missense variant [p.(Ser327Asn)] was transmitted in an X-linked recessive manner in a family with five affected males and two healthy carrier females^11^. These five males showed ID (5/5), several facial dysmorphisms [large nose (5/5), prominent ears (5/5), frontal baldness (4/5)], hearing loss (3/5), short stature (5/5), and cleft palate (1/5). An additional two hemizygous missense variants [p.(Tyr159Cys) and p.(Lys1009Asn)] were recently reported in two unrelated males^9,10^. They showed facial dysmorphisms (2/2), cleft lip and palate (1/2), pituitary gland abnormality (1/2), patent foramen ovale (1/2), hypotonia (2/2), DD (2/2), and ID (2/2), as seen in the above family. Male patients with missense variants exhibited milder mutant effects than those with truncated variants, as expected.

In conclusion, of the two female patients with *STAG2* variants, one showed a severe prenatal phenotype, while the other showed a mild pediatric phenotype. X inactivation was highly skewed in both cases. This phenotypic difference might depend on another factor, such as a modifier, that is yet to be found.

## Supplementary information

Supplementary Information

## Data Availability

The relevant data from this Data Report are hosted at the Human Genome Variation Database at 10.6084/m9.figshare.hgv.2891, 10.6084/m9.figshare.hgv.2894.

## References

[1] Kline AD (2018). Diagnosis and management of Cornelia de Lange syndrome: first international consensus statement. Nat. Rev. Genet..

[2] Leroy C (2016). Xq25 duplication: the crucial role of the STAG2 gene in this novel human cohesinopathy. Clin. Genet..

[3] Deardorff MA (2012). RAD21 mutations cause a human cohesinopathy. Am. J. Hum. Genet..

[4] Baquero-Montoya C (2014). Could a patient with SMC1A duplication be classified as a human cohesinopathy?. Clin. Genet..

[5] Yu L (2015). Increased burden of de novo predicted deleterious variants in complex congenital diaphragmatic hernia. Hum. Mol. Genet..

[6] Yingjun X (2015). Microduplication of chromosome Xq25 encompassing STAG2 gene in a boy with intellectual disability. Eur. J. Med. Genet..

[7] Mondal G, Stevers M, Goode B, Ashworth A, Solomon DA (2019). A requirement for STAG2 in replication fork progression creates a targetable synthetic lethality in cohesin-mutant cancers. Nat. Commun..

[8] Mullegama SV (2017). De novo loss-of-function variants in STAG2 are associated with developmental delay, microcephaly, and congenital anomalies. Am. J. Med. Genet. A.

[9] Mullegama SV, Klein SD, Signer RH, Vilain E, Martinez-Agosto JA (2019). Mutations in STAG2 cause an X-linked cohesinopathy associated with undergrowth, developmental delay, and dysmorphia: expanding the phenotype in males. Mol. Genet. Genom. Med..

[10] Yuan B (2019). Clinical exome sequencing reveals locus heterogeneity and phenotypic variability of cohesinopathies. Genet. Med..

[11] Soardi FC (2017). Familial STAG2 germline mutation defines a new human cohesinopathy. NPJ Genom. Med..

[12] Kruszka P (2019). Cohesin complex-associated holoprosencephaly. Brain.

[13] Berkovic SF (2019). The epilepsy genetics initiative: systematic reanalysis of diagnostic exomes increases yield. Epilepsia.

[14] Iwama K (2018). A novel SLC9A1 mutation causes cerebellar ataxia. J. Hum. Genet..

[15] Fromer M (2012). Discovery and statistical genotyping of copy-number variation from whole-exome sequencing depth. Am. J. Hum. Genet..

[16] Nord AS, Lee M, King MC, Walsh T (2011). Accurate and exact CNV identification from targeted high-throughput sequence data. BMC Genomics.

[17] Amos-Landgraf JM (2006). X chromosome-inactivation patterns of 1,005 phenotypically unaffected females. Am. J. Hum. Genet..

