# Supplementary material for: Nonsense variants of STAG2 result in distinct congenital anomalies
Source: Hum Genome Var. 2020 Sep 18;7:26. doi: 10.1038/s41439-020-00114-w (PMC7501222; doi:10.1038/s41439-020-00114-w)
Supplement: Supplementary file 1 — Supplementary Information [file 41439_2020_114_MOESM1_ESM.docx]

Table S1. *De novo* variants in Case 1

| Gene | Genomic change (hg19) | gDNA change | Protein change | Exon | *Inheritance* | ExAC | In house | SIFT | PROVEAN | Polyphen-2 | CADD |
| --- | --- | --- | --- | --- | --- | --- | --- | --- | --- | --- | --- |
| *MUC3A* | Chr7:100550582T>G | c.1163T>G | p.(Val388Gly) | Exon2 | *de novo* | 0 | 0 | Torelated | Neutral | Benign | 4.722 |
| *GPR180* | Chr13:95275451T>C | c.983T>C | p.(Val328Ala) | Exon7 | *de novo* | 0 | 0 | Torelated | Neutral | Benign | 7.499 |
| *STAG2* | ChrX:123220440C>T | c.3097C>T | p.(Arg1033*) | Exon30 | *de novo* | 0 | 0 | N/A | N/A | N/A | 46 |

ExAc, ExAc browser (http://exac.broadinstitute.org/); In house, in houses 575 Japanese control exome datasets; SIFT, Sorting Intolerant From Tolerant (http://sift.bii.a-star.edu.sg/)

PROVEAN, PROVEAN human genome variants (http://provean.jcvi.org/index.php); PolyPhen-2, Polymorphism Phenotyping v2 (http://genetics.bwh.harvard.edu/pph2/)

CADD, Combined Annotation Dependent Depletion (https://cadd.gs.washington.edu/)

Case 2

Case 1

Figure S1. X-inactivation studies using the HUMARA assays. X-inactivation was highly skewed in the patients.

The paternal and maternal X chromosome were inactivated in Cases 1 and 2, respectively.


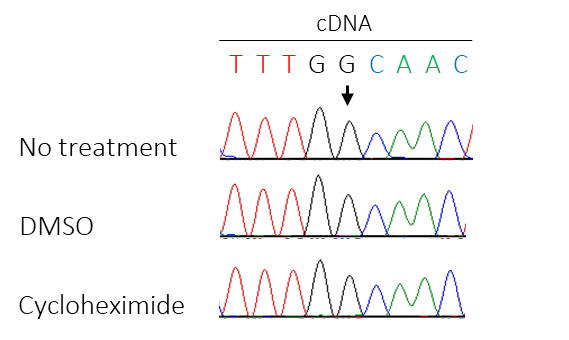


Figure S2. Electropherograms of RT-PCR sequencing in Case 2 with c.2229G>A. Arrow indicates that only the wild-type allele was transcribed. DMSO: vehicle control. Cycloheximide is used for the inhibition of nonsense-mediated mRNA decay.
